# Sodium hydrosulfide mitigates dexamethasone‐induced osteoblast dysfunction by interfering with mitochondrial function

**DOI:** 10.1002/bab.1786

**Published:** 2019-06-24

**Authors:** Jun Ma, Qiang Fu, Zhu Wang, Peng Zhou, Suchi Qiao, Bo Wang, Aimin Chen

**Affiliations:** ^1^ Department of Orthopedic Trauma Surgery Changzheng Hospital, The Second Military Medical University Huangpu District Shanghai People's Republic of China; ^2^ Department of Trauma and Orthopedics YueYang Hospital Shanghai People's Republic of China; ^3^ Department of Trauma and Orthopedics Peking University People's Hospital Beijing People's Republic of China

**Keywords:** hydrogen sulfide, glucocorticoids, osteoporosis

## Abstract

Osteoporosis is one of the clinical complications of long‐term treatment with glucocorticoids (GCs), characterized by systemic damage of bone mass and osteoblast dysfunction. Hydrogen sulfide was found to be involved in GCs‐induced osteoblast dysfunction. Osteoblastic MC3T3‐E1 cell and mitochondrial function were determined by cell viability, M‐CSF level, and ALP activity and superoxide production, membrane potential, and ATP level, respectively. The purpose of this research was to explore the impact of NaHS on osteoblastic MC3T3‐E1 cell function as well as on Sirt1 and PGC1*α* expression in dexamethasone (DEX)‐treated osteoblast cells. DEX‐treated MC3T3‐E1 cells exhibited decreased cell viability and ALP activity, as well as increased M‐CSF level; all these changes were dramatically attenuated by NaHS. DEX‐treated cells also displayed mitochondrial dysfunction, namely decreased mitochondrial membrane potential and ATP generation and increased superoxide generation, which were partly reversed by NaHS. We confirmed decreased Sirt1 and PGC1*α* protein expression in DEX‐treated MC3T3‐E1 cells by Western blot, which was also partly reversed by NaHS. Silencing of Sirt1 abrogated the protective effect of NaHS against DEX‐induced cell damage and mitochondrial dysfunction. NaHS alleviates DEX‐induced osteoblastic MC3T3‐E1 cell injury by improving mitochondrial function.

## 1 Introduction

Osteoporosis is considered as a common clinical complication of long‐term treatment with glucocorticoids (GCs), which was characterized by systemic damage of bone mass and microarchitecture that leads to fragility fractures and could result in a significant medical and socioeconomic burden. Previous studies demonstrated that osteoblast dysfunction induced an impaired balance between bone formation and bone resorption in bone‐mass regulation that contributed to GCs‐induced osteoporosis (GIO), for example, repressed osteoblast proliferation, improved differentiation of human preosteoblasts and altered M‐CSF expression 1.

An increasing amount of research has indicated that oxidative stress exerts a critical role in GIO pathogenesis, which was partly attributed to the excessive generation of reactive oxygen species (ROS), leading to increased ROS‐mediated oxidative protein modification 2. Mitochondrial electron transport was considered as the main source of bone tissue. Previous studies demonstrated that excessive GCs induce massive production of detrimental mitochondrial ROS, which initiates mitochondrial oxidative injury and contributes to the pathogenesis of multiple diseases, for example, endotoxin cholangitis and neurodegeneration disease 3, 4. Moreover, extensive bodies of research have demonstrated that oxidative stress‐induced mitochondrial dysfunction affected the regulation of osteoblast function 5, 6. Therefore, it is of interest to determine whether oxidative stress‐mediated mitochondrial dysfunction contributes to GCs‐induced osteoblast dysfunction.

Hydrogen sulfide (H_2_S) as ‘‘the third endogenous gaseous signaling transmitter’’ plays a critical role in multiple diseases, including renal disease 7, Alzheimer's disease 8, hypertension 9, cardiovascular disease 10, and osteoporosis 11. Endogenous H_2_S is produced from l‐cysteine primarily through the activities of cystathionine‐b‐synthase (CBS) and cystathionine‐c‐lyase (CSE) 12. H_2_S has been shown to repress dexamethasone (DEX)‐induced osteoblast injury by activation of AMPK signaling 13. Xu et al. 11 demonstrated that H_2_S exerts a protective role in osteoblastic MC3T3‐E1 cells through inhibiting H_2_O_2_‐induced oxidative damage, which provided the idea for the treatment of osteoporosis.

Among many genes influencing mitochondrial function, a large number of studies have concurred in highlighting a fundamental role for Sirt1 and PGC1*α*. Sirt1‐PGC1*α* is a major regulator of mitochondrial function, and Sirt1 and PGC1*α* expression disorder‐mediated mitochondrial oxidative stress plays a critical role in numerous diseases 14, 15, 16, 17.

In the present study, we examine CBS and CSE expression in DEX‐treated osteoblastic MC3T3‐E1 cells and the impact of exogenous H_2_S on DEX‐induced osteoblast injury. Then, we investigate mitochondrial function and the change of Sirt1 and PGC1*α* to determine whether regulation of mitochondrial function is involved in the protective effect of exogenous H_2_S against DEX‐induced osteoblast injury.

## 2 Materials and Methods

### 2.1 Cell culture and drugs administration

Murine osteoblastic MC3T3‐E1 cells were provided by the Chinese Academy of Sciences (Shanghai, China). Cells were cultured in *α*‐minimal essential medium (*α*‐MEM) containing 10% fetal bovine serum (FBS) at 37 °C under 5% CO_2_–95% air. DEXe 5 µM. NaHS (Sigma–Aldrich) was dissolved in sterile normal saline and used before the administration of DEX 24 H early. The control group was subjected to an equivalent volume of solvent.

### 2.2 Detection of mitochondrial superoxide production

MitoSOX is a novel mitochondrial fluorescent probe that specifically targets mitochondria, and thus selectively detects superoxide in mitochondria. Osteoblastic MC3T3‐E1 cells were subjected to dimethyl sulfoxide (DMSO)‐dissolved MitoSOX (Thermo Fisher) at a final concentration of 5 µM with the DMSO diluted to <0.1% for 10 Min at 37 °C in the dark. Following three washes with working buffer, red fluorescence was determined at 510/580 nm excitation/emission using a Synergy TM fluorescence plate reader (Bio‐Tek Instruments).

### 2.3 Detection of mitochondrial membrane potential

JC‐1 (Sigma–Aldrich) can serve as a fluorescent ratiometric probe to detect mitochondrial membrane potential change in cells, tissues, and isolated mitochondria. J‐aggregates were formed in the mitochondrial matrix and generated red fluorescence with high membrane potential. Conversely, JC‐1 monomer cannot accumulate in the mitochondrial matrix and produces green fluorescence with low membrane potential. The relative ratio of red to green fluorescence was used to indicate the proportion of mitochondrial depolarization, and thus indirectly reflects the mitochondrial function. Murine osteoblastic MC3T3‐E1 cells were subjected to 2 µM JC‐1 for 15 Min. Following dyeing, the excitation/emission signal of the J‐aggregates was 530/630 nm and the excitation/emission signal of the JC‐1 monomer was 488/530 nm using a SynergyTM fluorescence plate reader 18.

### 2.4 Measurement of ATP concentration

ATP concentration measurement was performed using an ATP Assay Kit (Beyotime) in accordance with manufacturer's instructions. Briefly, osteoblastic MC3T3‐E1 cells were homogenized in Lysis Buffer. The supernatant obtained after centrifugation at 12,000*g* for 5 Min was used to determine the ATP concentration using an ATP bioluminescence assay. The signal emitted from a luciferase‐mediated reaction was detected using a tube luminometer (Tecan) 18.

### 2.5 RNA interference *in vitro*

Sirt1 siRNA was designed and synthesized by GenePharma Corporation (Shanghai, China). Sirt1 siRNA sequence was: Forward: 5′‐GAAGU UGACC UCCUCA UUGUdT dT‐3′; Reverse: 5′‐ACAAU GAGGA GGUCA ACUUC dTdT‐3′. The negative control siRNA sequence was as follows: 5′‐TTCTCCGAACGTGTCACGT‐3′. SiRNA transfection in osteoblastic MC3T3‐E1 cells was performed using Xfect™ RNA Transfection Reagent (Clotech, Takara) in accordance with the manufacturer's instructions.

### 2.6 MTT test

The MTT test was performed to evaluate the proliferation of osteoblastic MC3T3‐E1 cells. Osteoblastic MC3T3‐E1 cells were cultured in a 96‐well cell culture plate, and 0.5 mg/mL MTT was added into each well and further incubated for 2–4 H after drugs treatment. The generated formazan crystals were dissolved in DMSO and the absorbance determined at 550 nm using a microplate reader (Tecan).

### 2.7 ALP activity assay

Osteoblastic MC3T3‐E1 cells were cultured in *α*‐MEM containing 10% FBS, and after drugs treatment 1% Triton X100 was added into the medium. The medium was centrifuged at 14,000*g* at 4 °C for 20 Min to recover the supernatant. The method of Bowers and McComb was performed to measure the change in absorbency at 405 nm.

### 2.8 Alizarin red S (ARS) staining

Cells were seeded onto six‐well plates at 1 × 10^5^ cells per well. Following cell culture with differentiation medium for 14 days, the cells were subjected to ARS staining. Briefly, the cells were washed three times with phosphate‐buffered saline, fixed with 4% paraformaldehyde for 15 Min and stained with 0.2% ARS solution (Cyagen, Suzhou, China) for 30 Min at 37 °C. Staining was repeated at least three times independently.

### 2.9 Western blot analysis

Osteoblastic MC3T3‐E1 cells were lysed using cold RIPA (Beyotime) containing 1% Protease Inhibitor Cocktail (Thermo Fisher) to obtain total protein. A 10% SDS‐PAGE was performed to separate the proteins, which were transferred to PVDF membranes. After blocking with 5% skimmed milk and 0.1% TBST for 2 H, the membranes were incubated with Sirt1 (Santa Cruz), PGC1*α* (Abcam), or *β*‐actin (Santa Cruz) antibodies in antibody dilution buffer (Beyome) at 4 °C overnight. The membranes were incubated with a secondary horseradish peroxidase‐conjugated antibody for 1–2 H at room temperature. The enhanced chemiluminescence Western blotting detection system (Santa Cruz) and a GeneGnome HR scanner (SynGene) were used to visualize the immunoreactive proteins and chemiluminescent signal from the membranes, respectively.

### 2.10 Statistical analysis

Data are expressed as means ± standard error of the mean. One‐way analysis of variance was used to compare multiple groups and the Student–Newman–Keuls test was used to perform comparisons between each group when significant (*P* < 0.05). Data analyses were performed with SPSS 16.0. *P* < 0.05 was considered statistically significant.

## 3 Results

### 3.1 DEX decreases osteoblasts CSE and CBS expression and H_2_S production

**Figure 1:**
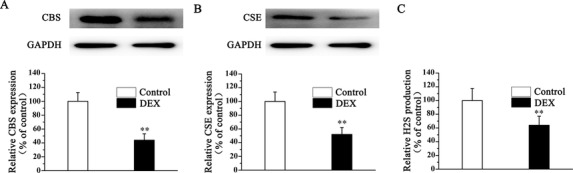
DEX decreases osteoblasts CSE, CBS expression, and H2S production. Cells were subjected to the presence of 1 mM DEX for 48 H. The expression of CBS (A), CSE (B), and H2S (C) generation in osteoblastic MC3T3‐E1 cells was assessed. All bar graphs represent means ± SEM (*n* = 4). **P < 0.01 versus control.

To demonstrate the role of H_2_S in the process of DEX‐induced osteoblast dysfunction, we first determined the expression of two main H_2_S‐producing enzymes, CBS and CSE, in osteoblastic MC3T3‐E1 cells. DEX significantly decreased CBS and CSE expression (Figs. 1A and 1B). Moreover, H_2_S production was also dramatically inhibited in DEX‐treated osteoblastic MC3T3‐E1 cells (Fig. 1C), which indicated that H_2_S might contribute to DEX‐induced osteoblast dysfunction.

### 3.2 NaHS mitigates DEX‐induced osteoblast dysfunction

**Figure 2:**
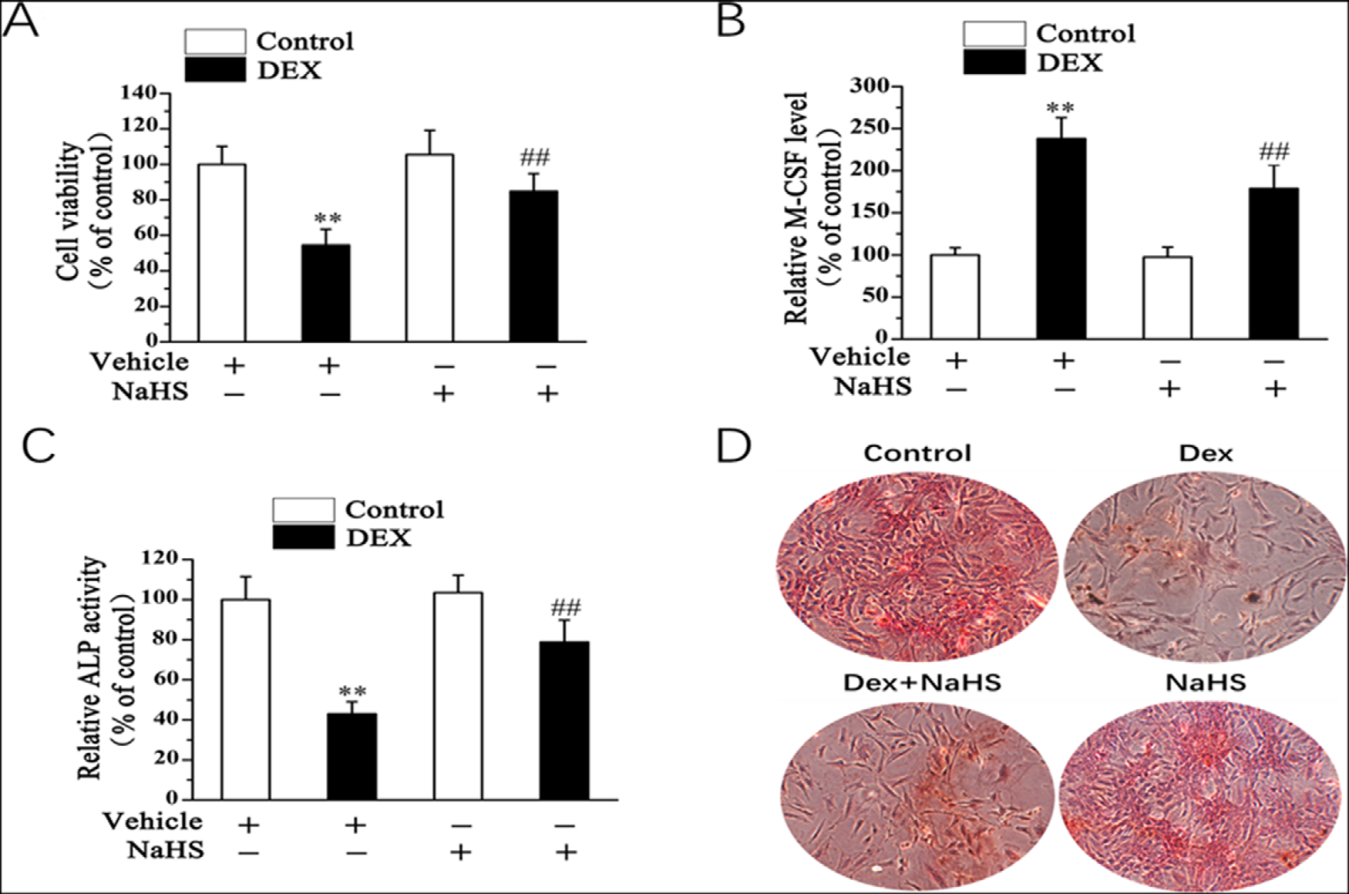
NaHS mitigates DEX‐induced osteoblast dysfunction. 20 µM saline or NaHS was added to osteoblastic MC3T3‐E1 cells. Twenty‐four hours later, cells were subjected to the presence of 1 mM DEX for 48 H. Cell vitality (A), the level of M‐CSF (B), and ALP activity (C) were assessed. (D) As shown by ARS staining (day 14) (x40), NaHS attenuates DEX‐inhibited osteogenic differentiation in osteoblasts. All bar graphs represent means ± SEM (*n* = 4). **P < 0.01 versus control; ^##^ *P* < 0.01 versus DEX.

To further investigate whether exogenous H_2_S was involved in the DEX‐induced osteoblast dysfunction, NaHS was used to treat osteoblastic MC3T3‐E1 cells. In DEX‐treated osteoblastic MC3T3‐E1 cells, cellular viability was significantly inhibited and the M‐CSF level was increased (Figs. 2A and 2B). Moreover, the level of ALP activity was decreased (Fig. 2C). NaHS treatment of DEX‐treated cells resulted in a significant increase in proliferation and the ALP activity and a decrease in the M‐CSF level (Figs. 2A–2C). Furthermore, DEX decreased osteogenic differentiation, which was attenuated by NaHS, as evidenced by ARS staining (Fig. 2D). Taken together, these results strongly suggested that NaHS mitigates DEX‐induced osteoblast injury.

### 3.3 NaHS mitigates DEX‐induced osteoblast mitochondrial dysfunction

**Figure 3:**
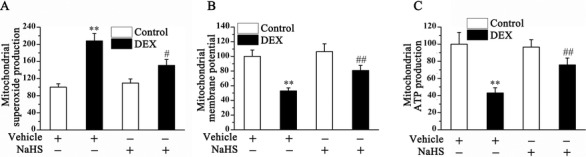
Effect of NaHS on mitochondrial function in control and DEX‐treated osteoblastic MC3T3‐E1 cells. The superoxide production (A), membrane potential (B), and ATP production (C) in mitochondria were measured in control and DEX‐treated osteoblastic MC3T3‐E1 cells. Bar charts represent means ± SEM (*n* = 4). **P < 0.01 versus control; ^#^ *P* < 0.05, ^##^ *P* < 0.01 versus NaHS–DEX.

Previous research demonstrated that mitochondrial dysfunction was involved in the regulation of osteoblast function 18. Therefore, we examined the mitochondrial function of DEX‐treated osteoblastic MC3T3‐E1 cells, including superoxide production, membrane potential, and mitochondrial ATP production. Mitochondria from DEX‐treated osteoblasts were found to display a dramatic increase in mitochondrial superoxide production (Fig. 3A), as well as decreases in mitochondrial membrane potential (Fig. 3B) and ATP production (Fig. 3C). NaHS administration reversed these DEX‐induced mitochondrial‐related changes in osteoblastic MC3T3‐E1 cells, indicating NaHS mitigates DEX‐induced osteoblast mitochondrial dysfunction.

### 3.4 Involvement of Sirt1 and PGC‐1*α* in the protective effect of H_2_S against DEX‐induced osteoblast mitochondrial dysfunction

**Figure 4:**
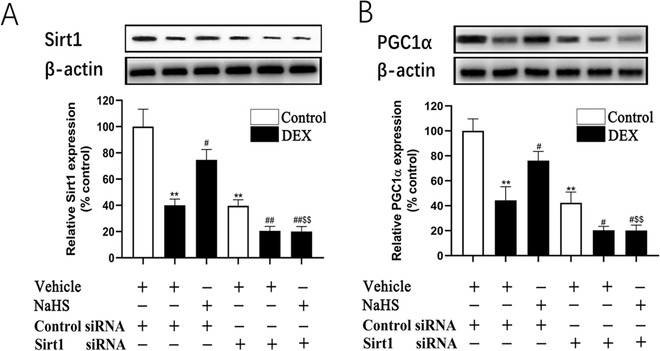
Sirt1 siRNA attenuates the protective effect of NaHS on DEX‐associated lower expression of Sirt1 and PGC1α. Western blot analysis was performed to assess the expression of Sirt1 (A) and PGC1α (B) in protein level. Bar charts represent means ± SEM (*n* = 4). *P < 0.05, **P < 0.01 versus control; ^#^ *P* < 0.05, ^##^ *P* < 0.01 versus DEX; ^$^ *P* < 0.05, ^$$^ *P* < 0.01 versus NaHS–DEX.

**Figure 5:**
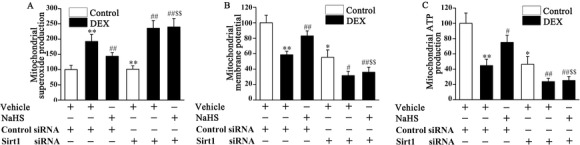
Sirt1 siRNA attenuates the protective effect of NaHS on DEX induced mitochondrial dysfunction in osteoblastic MC3T3‐E1 cells. The mitochondrial function was assessed by superoxide production (A), membrane potential (B), and mitochondrial ATP generation(C) in mitochondria. Bar charts represent means ± SEM (*n* = 4). *P < 0.05, **P < 0.01 versus control; ^#^ *P* < 0.05, ^##^ *P* < 0.01 versus DEX; ^$$^ *P* < 0.01 versus NaHS–DEX.

Sirt1 and PGC1*α* play an important role in mitochondrial function. Both Sirt1 and PGC1*α* protein expression were significantly decreased in DEX‐treated osteoblastic MC3T3‐E1 cells, which could be reversed by NaHS treatment (Figs. 4A and 4B). To determine the role of Sirt1 in osteoblast injury, siRNA was performed to knock down Sirt1 expression in the osteoblast. Sirt1 siRNA caused an approximately 80% decrease in Sirt1 expression in osteoblastic MC3T3‐E1 cells (Fig. 4A). Furthermore, Sirt1 siRNA blocked NaHS‐induced upregulation of Sirt1 expression in DEX‐treated osteoblastic MC3T3‐E1 cells. More interesting was that NaHS‐induced PGC1α expression in DEX‐treated osteoblastic MC3T3‐E1 cells was also dramatically repressed by Sirt1 siRNA (Fig. 4B). The protective effect of NaHS against DEX‐induced mitochondrial damage was blocked by Sirt1 siRNA, as evidenced by an increase in mitochondrial superoxide production (Fig. 5A) and by distinct decreases in mitochondrial membrane potential (Fig. 5B) and ATP production (Fig. 5C) in DEX‐treated osteoblastic MC3T3‐E1 cells.

### 3.5 NaHS protects against DEX‐induced osteoblast injury via Sirt1

**Figure 6:**
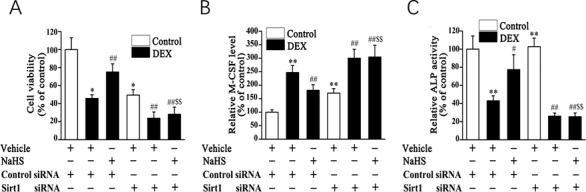
Sirt1 siRNA attenuates the protective effect of NaHS on DEX induced injury in osteoblastic MC3T3‐E1 cells. Cell viability (A), the level of M‐CSF (B), and ALP activity (C) were assessed. Bar charts represent means ± SEM (*n* = 4). *P < 0.05, **P < 0.01 versus control; ^#^ *P* < 0.05, ^##^ *P* < 0.01 versus DEX; ^$$^ *P* < 0.01 versus NaHS–DEX.

We also found that Sirt1 siRNA could block the protective impact of NaHS against DEX‐induced osteoblast injury, as evidenced by increased cell proliferation (Fig. 6A) as well as a decreased level of M‐CSF (Fig. 6B) and an increased level of ALP activity (Fig. 6C) in the cell‐culture medium of DEX‐treated osteoblastic MC3T3‐E1 cells.

## 4 Discussion

Osteoporosis is a common clinical disease characterized by bone mass loss that is attributed to an impaired balance between bone formation and bone resorption. Bone formation is a complex but well‐organized process, in which osteoblasts play an important role. Considerable research has focused on the factors that may promote bone formation or affect the proliferation and differentiation of osteoblasts. For example, bone morphogenetic protein‐2 can promote bone formation and has been combined with various delivery carriers in bone regeneration practice 19, 20. The role of the mechano‐growth factor (MGF) Ct24E was investigated in bone formation and remodeling, which showed that MGF‐Ct24E has a marked ability to increase bone formation by increasing cell proliferation and delaying cell differentiation 21. Li et al. 22 reported that B‐cell maturation antigen plays a regulatory role on the toxic effect of chromium ions on human SaOS‐2 osteoblasts. In the present study, we demonstrated that DEX inhibited osteoblastic differentiation of osteoblastic MC3T3‐E1 cells. Furthermore, DEX decreased CBS and CSE expression in these cells. Moreover, NaHS, an H_2_S donor, alleviated DEX‐induced inhibition of osteoblastic differentiation of MC3T3‐E1 cells and their CBS/CSE expression. NaHS also significantly reversed DEX‐induced oxidative stress and mitochondrial dysfunctions in MC3T3‐E1 cells.

In clinical, prolonged and/or overdose administration of GCs, many side‐effects are displayed, including hypertension, hyperglycemia, glaucoma, osteonecrosis, and osteoporosis. GCs are known to impair osteoblast function and inhibit osteoblast differentiation. For example, DEX can inhibit cell differentiation in osteoblastic OB‐6 cells 23. Similarly, in the present study, we found that DEX could inhibit cell viability of MC3T3‐E1 cells in a dose‐dependent manner.

It was reported that mitochondria play an important role in many biological processes. For example, recent studies have demonstrated that normal mitochondrial function contributes to osteogenic differentiation 24, 25. Kato et al. 26 reported that decreased mitochondrial function caused impaired osteoblast differentiation. Weili et al. 27 suggested that Scolopin‐2‐NH2 interacted with mitochondria and could play an important role in the apoptosis process. The toxic effects produced by GCs were demonstrated to be involved in mitochondrial dysfunction 28. In the present study, GCs reduced ATP production in mitochondria and MMP of MC3T3‐E1 cells, whereas mitochondrial superoxide production was increased.

H_2_S is a novel gasotransmitter endogenously produced by mammalian tissues and mediates diverse physiological functions. Recent studies have demonstrated the cytoprotective activity of H_2_S in a number of systems 29, 30, 31. For example, H_2_S was found to be a protective molecule against oxidative stress by restoring redox homeostasis and inducing the antioxidant transcription factor Nrf2. Additionally, H_2_S acts as an anti‐inflammatory agent and protects against leukocyte‐mediated inflammation. Furthermore, H_2_S inhibits rotenone‐induced neuronal cell apoptosis via preservation of mitochondrial function. In osteoblastic‐like MC3T3‐E1 cells, H_2_S protects against oxidative stress via inhibition of mitogen‐activated protein kinase signaling 13. In the present study, we also found that NaHS administration could alleviate DEX‐induced inhibition of osteoblastic differentiation of MC3T3‐E1 cells via the maintenance of mitochondrial function.

Sirt‐1 and PGC‐1*α* are known to be important regulators in maintaining mitochondrial function 32. However, no convincing data have been presented to date for the mechanisms of its bone‐protective action. PGC1*α* serves as a central regulator of mitochondrial function through the activation of mitochondrial energy metabolism, respiration, and biogenesis 33. Moreover, ever more research has demonstrated that Sirt1 together with PGC‐1*α* exert a role in various diseases, for example, myocardial ischemia/reperfusion‐induced oxidative injury in mitochondria 34, 35. In the present study, we observed an inhibitory effect of DEX on Sirt1/PGC‐1*α* protein expression in MC3T3‐E1 cells, which could be mitigated by NaHS administration. Interestingly, NaHS‐induced increased PGC1*α* expression in MC3T3‐E1 cells was abolished by Sirt1 knockdown. Moreover, the beneficial impact of NaHS against DEX‐associated mitochondrial injury and cell damage was also reversed by Sirt1 knockdown. Collectively, we suggest that PGC1*α* may be simultaneously modulated by two post‐transcriptional pathways: Sirt1‐dependent activation and mitochondrial oxidative stress‐dependent inhibition. In NaHS‐treated MC3T3‐E1 cells, Sirt1 upregulation and a decrease of mitochondrial oxidative stress‐dependent inhibition led to increased PGC1*α* expression. Therefore, Sirt1 might be the pivotal molecule involved in the protective effect of NaHS against DEX‐induced MC3T3‐E1 cell damage. Given the crucial protective role of Sirt1 in DEX‐treated MC3T3‐E1 cells, the mechanism underlying the bone‐protective effect of H_2_S may converge on Sirt1. Future studies should examine the mechanism of Sirt1 in H_2_S‐induced improvement of DEX‐inhibited osteoblast differentiation.

## 5 Conclusions

Herein, we show that H_2_S protected against the inhibition of osteoblast differentiation in DEX‐treated MC3T3‐E1 cells. The evidence for improvement in cellular mitochondrial function suggests that the mitochondria‐protective action of H_2_S might partly contribute to ameliorating inhibition of osteoblast differentiation. These findings suggest that exogenous H_2_S is a promising option for the prevention and treatment of GC‐induced osteoporosis and osteonecrosis.

The authors declared no potential conflicts of interest with respect to the research, authorship, and/or publication of this article.
